# The role of G-CSF neuroprotective effects in neonatal hypoxic-ischemic encephalopathy (HIE): current status

**DOI:** 10.1186/s12974-021-02084-4

**Published:** 2021-02-21

**Authors:** John Sieh Dumbuya, Lu Chen, Jang-Yen Wu, Bin Wang

**Affiliations:** 1 Department of Pediatrics, Zhujiang Hospital of Southern Medical University, Guangzhou, 510282 People’s Republic of China; 2 Department of Biomedical Science, Charles E. Schmidt College of Medicine, Florida Atlantic University, Boca Raton, FL USA

**Keywords:** Hypoxic-ischemic encephalopathy, Neonatal, Pro-inflammatory cytokine, Apoptosis, Hypoxia ischemia, Granulocyte-colony stimulating factor, Angiogenesis, Neurogenesis

## Abstract

Hypoxic-ischemic encephalopathy (HIE) is an important cause of permanent damage to central nervous system (CNS) that may result in neonatal death or manifest later as mental retardation, epilepsy, cerebral palsy, or developmental delay. The primary cause of this condition is systemic hypoxemia and/or reduced cerebral blood flow with long-lasting neurological disabilities and neurodevelopmental impairment in neonates. About 20 to 25% of infants with HIE die in the neonatal period, and 25-30% of survivors are left with permanent neurodevelopmental abnormalities. The mechanisms of hypoxia-ischemia (HI) include activation and/or stimulation of myriad of cascades such as increased excitotoxicity, oxidative stress, N-methyl-d-aspartic acid (NMDA) receptor hyperexcitability, mitochondrial collapse, inflammation, cell swelling, impaired maturation, and loss of trophic support. Different therapeutic modalities have been implicated in managing neonatal HIE, though translation of most of these regimens into clinical practices is still limited. Therapeutic hypothermia, for instance, is the most widely used standard treatment in neonates with HIE as studies have shown that it can inhibit many steps in the excito-oxidative cascade including secondary energy failure, increases in brain lactic acid, glutamate, and nitric oxide concentration. Granulocyte-colony stimulating factor (G-CSF) is a glycoprotein that has been implicated in stimulation of cell survival, proliferation, and function of neutrophil precursors and mature neutrophils. Extensive studies both in vivo and ex vivo have shown the neuroprotective effect of G-CSF in neurodegenerative diseases and neonatal brain damage via inhibition of apoptosis and inflammation. Yet, there are still few experimentation models of neonatal HIE and G-CSF’s effectiveness, and extrapolation of adult stroke models is challenging because of the evolving brain. Here, we review current studies and/or researches of G-CSF’s crucial role in regulating these cytokines and apoptotic mediators triggered following neonatal brain injury, as well as driving neurogenesis and angiogenesis post-HI insults.

## Background

**Fig. 1:**
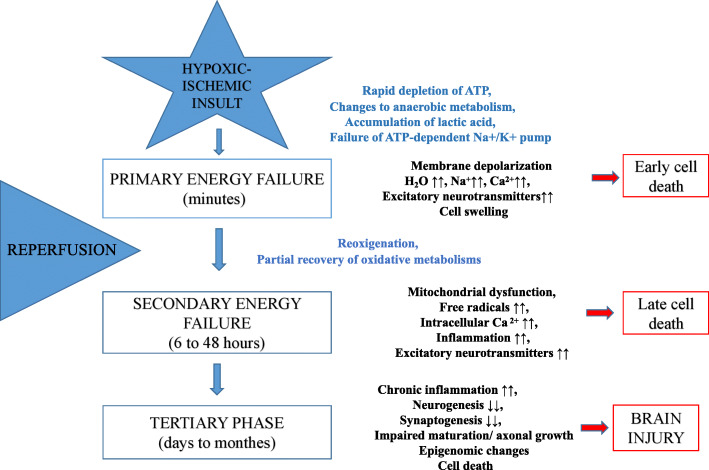
Schematic illustration of pathogenic mechanisms of HIE following HI brain injury. Primary energy failure occurs immediately after the hypoxic–ischemic insult. After reperfusion, there is a secondary energy failure, which can extend in duration from 6 to 48 h. Brain injury (tertiary phase) continues to occur months to years after the injury resulting in decreased plasticity and reduced number of neurons. Latent period following resuscitation is ideal for interventions to decrease the impact of secondary energy failure. However, strategies are developed to attenuate tertiary brain damage which will expand the therapeutic window, substantially increasing the beneficial effects of neuroprotection in these infants and hence its impact on long-term outcome. The up arrows represent an increase while the down arrows show a decrease in the corresponding metabolite/process

Numerous studies have shown that the most common contributor to early neonatal mortality is birth asphyxia with prematurity, infections, and low birth weight being other major contributors [1]. Birth asphyxia leads to significant brain injury with about 20-25% of asphyxiated newborns with hypoxic ischemic encephalopathy (HIE) dying within the newborn period and another 25% developing long-term sequelae, most commonly, cerebral palsy, epilepsy, autism spectrum disorder, and sensory deficits [2]. These neurological insults result in significant short-term and long-term physical, emotional, and financial cost [3]. HIE has a myriad of etiologies commonly categorized into prenatal and perinatal [4]. The pathophysiological presentation of HIE has been extensively explored, including but not limited to activation of inflammatory agents, apoptotic cascades, excitotoxicity, activation of microglia and astrocytes, N-methyl-d-aspartic acid (NMDA) receptor hyperexcitability, mitochondrial impairment and oxidative stress, and delayed cell death post-HI [5–8], categorized into three phases outlined in Fig. 1 [1, 9].

The clinical manifestations of neonatal HIE are insidious because the immature brain is more resistant to injury from hypoxia-ischemia (HI) events due to lower cerebral metabolic rate, plasticity of immature central nervous system (CNS), and immaturity in the development of balance in the functional neurotransmitters [10]. Thus, neonates suffering from HIE will go unnoticed during the early stages of HI, rendering them more susceptible to secondary injury occurring 6 to 72 h after the initial insults (Fig. 1). Similarly, clinical assessments are sometimes inconclusive or vague due to the nature of disease status and presentation in these infants. Diagnostic guidelines have been set out in neonates with HIE by the American Academy of Pediatrics (AAP) and the American College of Obstetrics and Gynecology (ACOG) [4] for initial assessment and appropriate management strategies. Routine serum biomarkers, magnetic resonance imaging (MRI), and electroencephalogram (EEG) [11–15] are the most commonly used diagnostic tools in recognizing brain injury in neonate that helps guide timely intervention and assessment of treatment outcome and prognostication.

Potential neuroprotective strategies targeting different pathways leading to neuronal cell death in response to hypoxic-ischemic insult have been investigated, including hypothermia, erythropoietin, magnesium, allopurinol, xenon, melatonin, growth factors (G-CSF, SCF, Epo), barbiturates, statins, and stem cells in various animal models of neonatal HIE [2, 16–18]. Therapeutic hypothermia (TH) is the most widely used standard treatment in neonatal HIE [19, 20], by inhibiting inflammatory cascades, reduced production of reactive oxygen species, inhibited apoptosis, an endogenous neuroprotective effect, reduced concentrations of free radicals, and neurotransmitters such as glutamate, glutamine, GABA, and aspartate [17, 21, 22], while others report ineffectiveness and/or adverse effects [23–25]. For instance, Arteaga et al. [9] reported that about 50% of infants treated with TH had adverse outcomes, such as cognitive impairment. A randomized control trial (RCT) by Shankaran et al. [26] concluded that cooling for 120 h or to 32.0 °C, or both, may be deleterious. Subsequently, TH did not improve EEG recovery when cooling was extended from 72 to 120 h and that it further impaired neuronal survival [27]. Thus, other neuroprotective agents are being explored that offer promise either as a monotherapy or in combination with therapeutic hypothermia.

**Fig. 2:**
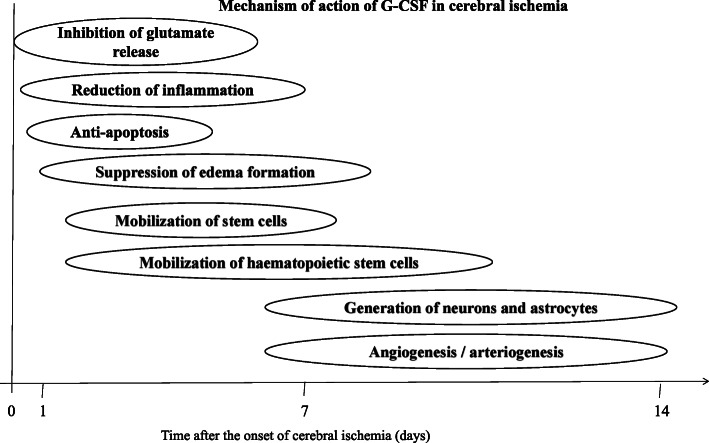
Schematic illustration of the potential mechanism of G-CSF action in hypoxia ischemia injury. In the acute phase of cerebral ischemia, G-CSF can protect the brain by inhibiting glutamate release, anti-inflammatory, anti-apoptotic, and inhibit edema formation. In subacute phase, GCSFR can stimulate endogenous neuronal regeneration, mobilization of bone marrow stem cells, driving neuronal regeneration and functional repair, and promote neovascularization (angiogenesis and neurogenesis) during the chronic phase

Granulocyte-colony stimulating factor (G-CSF) is a 20-kDa protein that readily crosses the blood-brain barrier (BBB), which is a member of the hematopoietic growth factor family, that promote the proliferation, differentiation, and survival of hematopoietic stem cells with the obvious protective effect on neurons in peripheral and central nervous system [28–30]. The past few decades have explored its role from neutropenia-induced chemotherapy to neurological diseases and traumatic brain injury through regulation of both inflammatory and apoptotic mediators and enhancing neurogenesis and angiogenesis (Fig. 2) [29, 31–34]*.* G-CSF and its receptors are expressed by neurons, and their expression is regulated by ischemia, which points to an autocrine protective signaling mechanism [35]. Extensive studies in both in vivo and ex vivo have shown the neuroprotective effect of G-CSF in neurodegenerative diseases (Table1) such as Parkinson’s disease [53, 54], Alzheimer’s disease [55], and stroke [56–58], and clinical trials are ongoing to determine its efficacy and safety in these neurological diseases [59, 60]. Recent studies have focused on its neuroprotective effect in neonatal brain injury by regulating inflammatory and apoptotic mediators, thus attenuating neuroinflammation and neuronal apoptosis as well as enhancing neurogenesis and angiogenesis. Indeed, there are current reviews on HIE that have detailed its pathogenesis [4, 27], diagnostic modalities [13, 15], treatment interventions [2, 17], and emerging therapeutic agents [1], including G-CSF. In addition to these reviews, ours mainly focused on G-CSF role in modulating inflammatory and apoptotic mediators, driving of neurogenesis and angiogenesis, and signalling pathways mediated by G-CSF in attenuating neonatal ischemic brain damage. We therefore review current studies and/or researches of G-CSF’s crucial role in regulating these cytokines and apoptotic cascades triggered following neonatal hypoxia-ischemia injury and subsequently its role in promoting neurogenesis and angiogenesis, thus shedding more light on the current understanding of G-CSF’s potential protective mechanism(s) in neonatal brain injury.

**Table 1:** An Overview of G-CSF application in Hypoxia-schaemia Brain Injury Models

Model	Adult/Neonate	Dosage (μg/kg)	Application/Duration	Outcome	Ref
Acute ischaemic cerebral injury (MCAO)	Adult SD rats	100	s.c. injected immediately after injury for 7d	Reduced infarct volume & necrotic cells	[29]
				Increased Egr-1 & VEGF expression levels	
Stroke (BCAO)	adult male Swiss Webster mice	50	s.c. injected 30min afterocclusion for 4 to 7 consecutive days	Increased expression of G-CSF receptor	[36]
				Decreased GRP78 expression & decreased ATF6 cleavage levels	
				Decreased apoptotic protein signalling (DRI1 & P53) & increased pro-survival signalling (OPAI)	
				Upregulated anti-apoptotic protein Bcle-2 and downregulated pro-apoptotic proteins Bax & Bak	
				Increased locomotor sensitisation	
Cerebral ischaemia reperfusion (tMCAO)	Adult SD rats	50	s.c. injected 1h after restoring CBF for 5 consecutive days	Reduced infarct volume & oedema	[37]
				Improved neurological function & reduced apoptotic neurons	
				Downregulated the activation of the JNK apoptosis pathway	
Ischaemic brain injury (DHCA)	Newborn piglets	34	iv. 2h prior to inintiation of bypass	Reduced neuronal injury in the hippocampus	[38]
Focal cerebral ischaemia (MCAO)	Adult male SD rats	50	s.c. injected at the onset of reperfusion; 2nd injected at onset of reperfusion for 2d	Attenuated infarct volume & early neurological deficits	[39]
				Elevated STAT3 phosphorylation & nuclear Pim-1 expression	
				Increased expression of cIAP2 & Bcl-2 & decreased caspase-3 & Bax levels	
Hypoxia-ischaemia (RCCA ligation)	Neonatal SD rats	50	s.c. injected 1h after HI for 56	Less vacuolization, neuron loss & tissue breakqown	[33]
				Increased brain weight & G-CSF receptor expression	
				Reduced cleaved caspase-3 activity	
				Increased expression of anti-apoptotic pathway mediators	
Hypoxic-ischaemic brain damage (RCCA ligation)	Neonatal SD rats	50	s.c. injected 1h after HI for 6 days & 11 days	Promoted physical development & improved functional deficits	[40]
				Reduced brain atrophy & increased systemic organ weight	
				Increased exploratory behaviour & shorm-term memory	
Hypoxia- ischaemia (RCCA ligation)	Neonatal SD rat pups	50	s.c. injected 1h after HI	Reduced infarct volume & corticosterone levels	[41]
				Decreased cleaved caspase-3 level & lowered Bax/Bcl-2 ratio	
				G-CSF did not influence ACTH response	
Hypoxia- ischaemia (RCCA ligation)	Neonatal SD rat pups	50	s.c. or i.p. 1h after HI for 4d	Reduced infarct volume & lung injury	[30]
				Increased neutrophil count & less brain tissue atrophy	
				Improved physical development & neurological function	
Hypoxia-ischaemia (RCCA ligation)	Neonatal SD rat pups	50	s.c. injected 1h after HI	Reduced infarct volume & increased expression of G-CSF receptor in neurons	[42]
				Increased p-AKt expression & decreased p-GSK-3β/GSK-3β ratio	
				Decreased aopototic markers & TUNEL positice cells in neuron	
Perinatal hypoxia	Neonatal SD rat pups	10, 30, 50	s.c. injected 1d after HI	Attenuated PSD-95 protein expression levels & improved long-term deficits	[43]
				Increased phosphorylated activity of pRaf-pERK1/2-PCREB pathway	
				Enhanced increase expression of neurogenesis in hippocampal neuron	
Stroke (MCA ligation)	Adult male SD rats	15	s.c. injected 1h after restoring CBF for 15d	Decreased mortality rate & less effect in reducing infarct volume	[44]
				Improved functional recovery of motor function	
				Increased number of proliferating cells & new neurons in the SVZ	
Hypoxia-ischaemia (RCCA ligation)	Neonatal SD rats	50	i.p. immediately after HI induction	Attenuated cerebral infarction & improved body weight	[45]
				Inhibited apoptosis by decreasing apoptotic cells & increased brain volume	
Focal cerebral ischaemia (MCAO)	Male Wistar rats	60	iv 30min after occlusion	Reduced infarct volume & mortality rate	[46]
				Increased STAT3 expression & anti-excitotoxic effect	
Stroke (MCAO/CCA)		50	iv 60min after induction	Elevated neutrophil count & reduced infarct volume	[47]
				Increased expression of G-CSF receptor in neurons	
(MCAO)		60	iv 2h after onset of occlusion for 5d	Increased protein level of STAT3 & increased AKt phosphorylation	
				Improved long-term behaviour	
Hypoxic-ischaemic brain injury (MCAO)	Neonatal mice pups	200	s.c. injected 1h after injury & 60h after injury for 5d	Did not improved neurobehavioural outcomes & brain injuries	[48]
Perinatal hypoxia	Neonatal rat pups	30	i.p. 1d after HI induction for 6d	Enhanced neurogenesis & improved long-term cognitive function	[49]
Hypoxia- ischaemia (RCCA ligation)	Neonatal SD rat pups	50	i.p. 2.5h after HI induction	Decreased expression levels of TNF-α and IL-1β & increased IL-10 levelsDecreased expression levels of TNF-α and IL-1β & increased IL-10 levels	[50]
				Increased Bcl-2 expression levels & decreased CC3 and Bax expression levels	
				Upregulated p-mTOR and p-P70S6K protein expression levels	
Hypoxia-ischaemia (RCCA ligation)	Neonatal SD rat pups	50	s.c. injected 1h after HI	Showed localisation of G-CSF receptor in enthothial cells	[51]
				Decreased β-catenin and p120-catenin phosphorylationDecreased β-catenin and p120-catenin phosphorylation	
				Attenuated PICs (IKKβ, NF-κB, TNF-α, IL-1β) & enhanced IL-10 levelsAttenuated PICs (IKKβ, NF-κB, TNF-α, IL-1β) & enhanced IL-10 levels	
				Decreased adheren proteins & increased tight junction proteins expression	
Hypoxia-ischaemia (RCCA ligation)	Neonatal SD rat pups	50	s.c. injected 1h after hypoxia	Inhibited corticosterone synthesis by activating its receptor in cortical cells	[52]
				Increased expression of JAK2, PI3K, AKt and PDE3B proteins	
				Inhibited cAMP elevation induced by cholera toxin	
				Decreased infarct volume and increased body weight	

*Abbreviations*: *G-CSF* Granulocyte-colony stimulating factor, *MCAO* Middle cerebral artery occlusion, *BCAO* Bilateral cerebral artery occlusion, *tMCAO* transient middle cerebral artery occlusion, *DHCA* Deep hypothermic circulation arrest, *RCCA* Right common carotid artery, *VEGF* Vascular endothelial growth factor, *HI* Hypoxia-ischaemia, *Egr-1* Early growth response-1, *s.c* subcutaneous, *iv* intravenous, *i.p* intraperitoneal, *GRP78* Glucose regulated protein 78, *ATF6* Activating transcription factor 6, *DRP1* Dynamin-related protein 1, *OPA1* Optic atrophy protein 1, *JNK* c-Jun N-terminal kinase, *Bcl-2* B-cell lymphoma 2, *cIAP2* cellular inhibitor of apoptosis protein 2, *ACTH* Adrenocorticotropic hormone, *p-GSK-3β* phosphorylated glycogen synthase kinase-3 beta, *p-AKt* phosphorylated protein kinase B, *PSD-95* Postsynaptic density protein-95, *pCREB* phosphorylated cAMP-responsive element binding protein, *pERK* phosphorylated exracellular signal-regulated kinase, *pRaf* phosphorylated mitogen-activated protein-kinase-kinase-kinase, *SVZ* Subventricular zone, *CC3* Cleaved caspase-3, *STAT3* Signal transducer and activated protein kinase 3, *p-P70S6K* Phosphorylated p70 ribosomal s6 protein kinase, *p-mTOR* Phosphorylated mammalian target of rapamycin, *IL-10* Interleukin 10, *IL-1β* Interleukin 1 beta, *TNF-α* Tumour necrosis factor-alpha, *NF-κB* Nuclear factor-kappa B, *IKKB* Inhibitor of kappa B kinase, *PICs* Pro-inflammatory cytokines, *PDE3B* Phosphodiesterase 3B, *AKt* Protein kinase B, *PI3K* Phosphatidylinositol 3-kinase, *JAK2* Janus kinase 2, *cAMP* cyclic adenosine monophosphate, *CBF* Cerebral blood flow

## Regulation of cytokines and apoptosis by G-CSF in neonatal HIE

There are numerous studies that have shown the neuroprotective effect of G-CSF via inhibition of apoptosis and inflammation as well as by stimulating angiogenesis and neurogenesis both in adults and neonatal animal HI models [61–64]. Indeed, G-CSF is implicated in the regulation of cytokines that are being disrupted following HI insults by decreasing pro-inflammatory and increasing anti-inflammatory cytokines. Similarly, G-CSF inhibits pro-apoptotic factors and increases anti-apoptotic factors.

### Regulation of cytokines by G-CSF in neonatal HIE

Prior studies have shown that interleukin-1 beta (IL-1ß) and tumor necrosis factor alpha (TNF-α) are early response cytokines in neuronal injury [65]. Increased expression of TNF-α induces neutrophil infiltration that increases endotheliocyte permeability and activates the matrix metalloproteinases (MMPs), which damage the blood-brain barrier (BBB) leading to swelling and degeneration of neurons and glial cells [66], while IL-1ß acts primarily through transcriptional activation of inducible nitric oxide synthase (iNOS) gene and nitric oxide (NO) generation thereby exacerbating brain injury [42]. These cytokines’ inflammatory response even persist in school-age children with neonatal encephalopathy (NE) showing poor neurodevelopmental outcome [48, 67]. Increased TNF-α expression also results in increase of caspase-3 cleavage and causes neuronal apoptosis [68]. Thus, TNF-α not only participates in neuronal inflammation but also in inducing apoptosis in neonatal HI injury. The imbalance between pro- and anti-inflammatory cytokines caused by HI damage also favors oligodendrocyte precursor proliferation into astrocytes instead of oligodendrocytes, and as a result, there is subsequent impairment of myelination, increased production of pro-inflammatory cytokines (PICs), and suppression of anti-inflammatory cytokine levels [69]. Activated astrocyte (astrogliosis) has both positive and negative roles in cytokine regulation following brain injury [70]; activated microglia have similar effects by expressing both pro-inflammatory and anti-inflammatory mediators depending on the degree and duration of insult being inflicted [4, 5, 71, 72].

The effect of G-CSF is demonstrated by preventing neuronal and glial pro-inflammatory cytokine expression. This is evidenced by preventing an overactivation of monocytes and lymphocytes by reducing the release of pro-inflammatory mediators while simultaneously activating the anti-inflammatory defense neutrophils [73]. Our previous study has shown that TNF-α and IL-1ß were upregulated after HI in neonatal rat model and were further elevated by rapamycin treatment with increased immunoreactivity of neuronal cell bodies. Their expression levels were subsequently decreased by G-CSF treatment [74]. Treatment with IL-10 is thought to decrease the level of TNF-α and IL-1ß after traumatic brain injury (TBI) [38]. Similarly, our previous study showed a decreased level of IL-10 expression after HI injury, while G-CSF administration was associated with its increased expression level thereby reducing further surges of TNF-α and IL-1ß and thus improving neurologic outcomes [74]. Xiao et al. [40] demonstrated that G-CSF treatment increased the mobilization of circulating CD34+ cells, polarizes T cell differentiation from Th1 to Th2 cells, and induces Th2 responses with the production of IL-4 and IL-10, accompanied by a decrease in production of IFN-ɣ and IL-2, thereby suppressing T cell proliferative responses to allogeneic stimulation that are anti-inflammatory and decreasing HI-induced injury, especially in the dentate gyrus by generating new neurons. G-CSF is said to significantly elevate the CD4 + CD25+ regulatory T cell subset in microglia-mediated reactive T cells as well as to inhibit MHC-II expression of microglia after lipopolysaccharide (LPS) activation or in the interactions of microglia and reactive T cells [75]. Other studies have shown that G-CSF effectively mobilized CD34-positive hematopoietic stem cells (HSC) in the preterm sheep thereby promoting proliferation of endogenous neural stem cells [76]. G-CSF can also stabilize the BBB and modulate neuroinflammation, and G-CSF is more neuroprotective in neonatal HI injury compared to adults [77].

Certain other studies have reported mixed results about the effectiveness of G-CSF treatment in ischemic stroke in terms of improving neurodegenerative and neurobehavioral outcomes, especially during the hyperacute and acute stage of HI injury [64, 78–80]. One study reported that G-CSF exacerbate brain damage as G-CSF increases the availability of neutrophils, which in turn enhances the inflammatory response in the brains of newborns [81]. Kallmunzer et al. [36] also reported a lack of neuroprotective or neuroregenerative effects of G-CSF in a rodent model of intracerebral hemorrhage. In a meta-analysis, England et al. [80] reported that G-CSF did not improve stroke outcome in individual patient with ischemic stroke when assessed by the National Institute of Health Stroke Scale (NIHSS) or Barthel Index (BI) while Huang et al. [64] analyzed 14 trials of G-CSF therapy in stroke and did not identify adequate evidence for the beneficial effects of this treatment modality in patients. Specifically, no favorable effects were noted on stroke outcomes including NIHSS score, the incidence of severe adverse events (SAEs), and mortality in patients treated with G-CSF versus control or placebo-treated patients. In another trial, the efficacy and tolerability of G-CSF were examined in patients with amyotrophic lateral sclerosis (ALS), a neurodegenerative disease, and the authors concluded that it has no clinical benefit in subjects with ALS [41], in contrast to previous studies [82, 83].

As some of these results and findings are mainly focused on adult ischemic stroke, it should be noted that differences do exist between the adult and neonate reaction to HI-induced brain insults, possibly due to immaturity of neonatal neurons. Specifically, there are differences in functional BBB response to acute experimental stroke between neonates and adults, as well as in gene expression of cerebral endothelial cells [25]. Other striking factors include initiation of G-CSF treatment, that is, the timing of G-CSF treatment might influence its neuroprotective effect; different dosages have been used both in ischemic stroke and neonatal HIE animal models, ranging from 10 to 250 μg/kg for subcutaneous injection and 5 to 60 mg/kg for intravenous injection [43, 61, 78]; route of administration varies according to each study; and duration of treatment ranges from 3 to 10 days [64, 80, 84]. Intranasal administration of G-CSF has also been examined in ischemic brain injury as potentially more effective and feasible [57]. All these factors influence the effectiveness of G-CSF across treatment modalities both in vivo and ex vivo. Therefore, such results should be judiciously interpreted and translated into neonates with HIE. Hence, there are still some loopholes and more studies are needed to examine the neuroprotective effect of G-CSF in neonatal brain injury at different times, doses, and duration in relation to regulation of neuroinflammatory and apoptotic cascades. Similarly, long-term neurofunctional assessment of G-CSF in neonatal hypoxia-ischemia brain injury should be warranted.

### Regulation of apoptosis by G-CSF in neonatal HIE

Apoptosis can be triggered via the extrinsic pathway, which involves activation of cell surface death receptors, or the intrinsic pathway, which requires mitochondrial outer membrane permeabilization (MOMP) [85], which leads to loss of mitochondrial transmembrane potential, formation of transition pores, and production of reactive oxygen species, subsequently leading to release of cytochrome c from mitochondria resulting in activation of caspases and other effectors of DNA fragmentation and cell death [7, 86, 87]. Increased expression of pro-apoptotic mediators causes translocation of apoptosis-inducing factor (AIF) from the mitochondria to the nucleus, where it interacts with DNA and stimulates chromatin condensation. Overexpression of AIF can aggravate neonatal brain injury after HI that further increases its translocation [39].

Studies have shown that neonatal HI injury is associated with Bax translocation to mitochondria with a concomitant decrease in BCL-2, resulting in activation of caspase-3 leading to apoptotic cell death that peaks from 24 to 72 h post HI brain injury [33]. BAD is involved in apoptotic and nonapoptotic processes, and these dual activities are regulated by post-transcriptional modifications. Activation of BAX and BAD also promote MOMP [88]. The BCL-2 family proteins are key regulators of MOMP and play critical role in the intrinsic apoptotic pathway, classified into anti-apoptotic (Bcl-2, Bcl-xl, Bcl-w) and pro-apoptotic (Bax, Bak, Bim, Bid, Bad) [45]. The increased expression of pro-apoptotic markers is also influenced by the hormones involved in the pituitary–adrenal response [89], while in the extrinsic pathway, binding ligands to death receptors (TNF-α, Fas, TRAIL, etc.) leads to activation of caspase-8 [49]. In addition, BCL-2 has been demonstrated to play a critical role in preventing apoptosis induced by rapamycin derivatives that have been approved for the treatment of patients with various malignancies, thus suggesting that overexpression of antiapoptotic proteins such as BCL-2 might serve as a surrogate marker for resistance to rapalogues [90]. GSK-3ß is highly expressed in brain regions including the cerebral cortex, hippocampus, and cerebellum, and its overactivation is involved in neuronal pro-apoptosis, and dysregulation of this kinase has a devastating effect on neurodevelopment [91]. Its overexpression can increase caspase-3 activity [92], which in turn activates apoptosis.

G-CSF administration downregulates GSK-3ß activity, resulting in reduced neuronal cell death, apoptosis, and infarct volume, as well as upregulating anti-apoptotic protein Bcl-2 expression levels [45, 77]. Our previous study has demonstrated that increased Bax expression levels and cleaved caspase-3 (CC3) activation were attenuated significantly by G-CSF treatment and simultaneously increasing BCL-2 expression levels that were decreased following HI-induced injury. We further stated that the effect of G-CSF in modulating these apoptotic factors was abolished by rapamycin, an inhibitor of mTOR [74]. Other studies have reported similar neuroprotective effects of G-CSF by inhibiting apoptosis [63]. The neuronal anti-apoptotic action of G-CSF may also be mediated in part by the anti-apoptotic protein cIAP2 [93]. G-CSF also inhibits the mitochondrial-dependent activation of caspase-3, an apoptotic activator during HI injury [94]. Thus, G-CSF’s underlying mechanism(s) in the neuronal anti-apoptotic effect can be indirect through suppression of TNF-α, which increases caspase-3 cleavage or directly inhibiting caspase-3 and other pro-apoptotic mediators.

## G-CSF in neurogenesis and angiogenesis

Neurogenesis is an important process for the reconstruction of neural networks and recovery of functional outcomes that are believed to continue throughout adulthood. It mainly occurs in the subventricular zone (SVZ) and subgranular layer of the hippocampal dentate gyrus, where the local environment tightly regulates neurogenesis [2]. Nonetheless, endogenous neurogenesis, stimulated by cerebral ischemia is not sufficient for the recovery of neurological functions [2]. The SVZ is more susceptible to neonatal HI brain injury located in the hippocampal dentate gyrus. Previous studies have shown that G-CSF facilitates bone marrow cell mobilization to the brain and drives neurogenesis and synaptic efficacy recovery thereby improving long-term functional outcome, which is mainly observed in the SVZ of the injured neonatal brain post-HI insult [61, 95, 96]. G-CSF also enhances concentrations of neurotrophic factors (GDNF and BDNF) that stimulate hippocampal neurogenesis as well as neuroplasticity by altering synaptic activity and possesses anti-apoptotic properties augmenting the neurogenic response to brain injury [97]. Endogenous role of G-CSF in the brain neuroprotective mechanism has also been demonstrated in ischemic model, where mice deficient of G-CSF showed overwhelming upregulation of matrix metalloproteinase 9 (MMP-9), a key factor in the activation of microglia and astrocytes, while treatment of G-CSF suppresses its expression [2, 98].

Angiogenesis is a physiological process by which new blood vessels are formed from pre-existing blood vessels. It is a complex and highly ordered process that relies upon extensive signaling networks both among and within endothelial cells (ECs) and their associated cells such as vascular endothelial growth factor (VEGF) proteins and angiopoietin-1 (Ang-1), which are required for angiogenesis [99]. Hypoxia-inducing factor-1 alpha (HIF-1α) is involved in early brain development and proliferation of neuronal progenitor cells. HIF also modulates cerebral hypoxic stress responses and activates endogenous neuroprotective systems during acute and late stages of HI damage of the developing brain [100]. HIF-1α induces VEGF expression and its receptors FMS-like tyrosine kinase (FLK-1) and fetal liver kinase-1 (Flk-1) in neurons facilitating blood reperfusion recovery, correlating with angiogenesis [25]. Late-stage of HIF-1α induction increases VEGF production that improves functional recovery and brain repair [101].

G-CSF administration increases local VEGF expression, which is necessary for vascular angiogenesis [102], thus corroborating its role in promoting angiogenesis through upregulation of VEGF expression via signaling pathways. Angiopeitin-1 (Ang-1), which is upregulated by G-CSF, is thought to reduce vascular solute permeability and contributes to vascular maturation and BBB stabilization by increasing the expression of BBB-related tight junction proteins (occludin, claudin-5, and zonula occludens protein 1) [103]. G-CSF treatment regulates the expression of VEGF and early growth response-1 (Erg-1) thereby ameliorating acute ischemic cerebral injury [29]. Mobilization of monocyte into blood vessels by G-CSF also stimulate angiogenesis [40]. Other reports argued that VEGF and MMP are involved in the initial opening of BBB within hours of an HI insult, which disrupt the basilar membrane and cause further damage to the BBB, especially VEGF-A which increases vascular permeability by uncoupling endothelial cell-cell junctions, resulting in BBB leakage and worsened outcomes [2, 103, 104]. Zhang et al. [25] stated that acute increases of VEGF results in the BBB leakage, whereas delayed upregulation of VEGF around the ischemic boundary area may prompt angiogenesis and reconstruction of the neurovascular unit (NVU). Thus, further research is needed to elucidate the pertinent role of VEGF after neonatal HI injury and its subsequent regulation by G-CSF.

## Combinational therapy with other agents

Recent reports have advocated for combination strategies in neonatal HI brain injury and that it is more effective than G-CSF monotherapy. For instance, Yu and colleagues [105] demonstrated that both erythropoietin (Epo) and G-CSF combined produce functional recovery in a mouse model of hypoxic-ischemic brain injury in a time-dependent manner, and the underlying mechanisms may be the induction of HIF-1α activity in both cytosol and nucleus, and an early change in the cell fate determination from astrogliosis toward neurogenesis. Erythropoietin (EPO) a glycoprotein that controls erythropoiesis is expressed in neural progenitor cells, mature neurons, astrocytes, oligodendrocytes, microglia, and endothelial cells. Epo has anti-apoptotic and anti-inflammatory effects and supports tissue remodeling by promoting neurogenesis, oligodendrogenesis, and angiogenesis [106]. Both G-CSF and EPO can readily cross the BBB, making them a possible candidate in neonates with HI insults. Liu et al. [107] reported that combination of G-CSF and EPO could synergistically promote proliferation of neural progenitor cells residing in the hippocampus and subventricular region of the brain. Another study examined the repetitive and long-term use of G-CSF+ EPO in stroke patients and reported similar neuroprotective effect with good tolerability and no associated adverse effects observed [108]. But in Yu and colleagues’ study, they reported that combined G-CSF and EPO does not show improvement during the chronic phase of HI injury [105]. Thus, the neuroprotective effects of combinational therapy of G-CSF and EPO in neonatal HIE need to be elucidated further to understand the underlying mechanisms.

Doycheva et al. [109] also concluded that G-CSF + SCF (stem cell factor) improved body weight, reduce brain-tissue atrophy, and improved neurological outcome following HI in the neonatal rat pup. Specifically, G-CSF + SCF induced more stable and long-lasting functional improvement in chronic strokes by increasing angiogenesis and neurogenesis through bone marrow-derived cells and the direct effects on stimulating neurons to form new neuronal networks [110, 111]. Hence, both G-CSF and SCF may work synergistically in improving the overall outcome of neonatal HI brain damage. Caspase-3 activation is reduced when G-CSF and SCF treatment are combined resulting in decreased apoptotic cell death, though other studies further reported that G-CSF/SCF and FL treatment did not affect apoptosis-inducing factor-dependent apoptosis or cell proliferation, and thus does not convey neuroprotection in neonatal HIE [112, 113]. Indeed, there are mixed reports about the neuroprotective effect of combinational G-CSF and SCF treatment in neonatal HI-induced brain injury. One study on HI mice demonstrated that G-CSF and SCF, given separately or in combination, have no neuroprotective effect, but rather a deleterious impact on neonatal excitotoxic brain damage [81].

G-CSF + hUCB can also decrease the number of MHCII+ cells not only in the corpus callosum and fornix but also in the cerebral peduncle. As G-CSF crosses the BBB, it acts upon neurons and glial cells through the G-CSF receptor. Indeed, glial cell activation has been demonstrated to downregulate expression of proinflammatory cytokines and to enhance neurogenesis [114]. One study demonstrated that intranasal administration of umbilical cord mesenchymal stem cells (UC-MSCs) significantly reduces neuroinflammation and protects hippocampal neurons, as well as increased concentration of the anti-inflammatory cytokine IL-10 in serum, thus contributing to neuroprotection [23]; similarly, UCB, especially its subtype EPCs (endothelial progenitor cells), has the ability to modulate neuroinflammation and reduce brain injury and behavioral deficits in perinatal HI brain injury [115]. Mesenchymal stem cells (MSCs) derived from human umbilical cord blood (hUCB) conferred neuroprotective and neuroregenerative benefits by improving angiogenesis and vasculogenesis [116]. MSCs plus G-CSF also decreased oxidative stress factors aggravated by HI brain injury [117]. G-CSF in combination with taurine is protective in primary cortical neurons against excitotoxicity induced by glutamate as well as suppresses endoplasmic reticulum (ER) stress [118]. Thus, synergistic therapies may exert and offer better functional improvements in neonatal HI brain injury, though more research is needed to explore the underlying mechanism(s).

Recently, Griva et al. [119] reported that the combination of neuroprotective treatments of G-CSF and enriched environment (EE) may enhance neuroprotection and it might be a more effective strategy for the treatment of neonatal hypoxic-ischemic brain injury by altering synaptic plasticity reflected by increased synaptophysin expression levels thus further enhancing cognitive function. Doycheva et al. [30] demonstrated that G-CSF + Ab improved body weight, reduced brain tissue damage, and improved long-term neurological function when assessed at 96 h and 5 weeks post HI in the neonatal rat pups, as well as conferring greater neuroprotection by depleting neutrophil accumulation, while G-CSF + metyrapone treatment not only lower caspase-3 expression level but also reduce corticosterone levels in neonates after HI injury [89]. Surprisingly, there has not been any study evaluating combinational therapy of G-CSF and therapeutic hypothermia (TH), as other modalities have been tried and underwent and/or are undergoing clinical trials in neonatal HIE, such as with epo and TH [120–122], xenon and TH [51, 123, 124], melatonin and TH [46, 47], and allopurinol and TH [125]. Thus, more study is needed to explore and evaluate the neuroprotective effect of G-CSF in combination with therapeutic hypothermia as well as other emerging agents.

## G-CSF-mediated signaling pathways in neonatal HIE

Stimulation of G-CSF by its receptor activates many downstream signaling pathways such as the Janus kinase (JAK)/signal transducer and activator of transcription (STAT), the Ras/mitogen-activated protein kinase (MAPK), and the phosphatidylinositol 3-kinase (PI3K)/protein kinase B (Akt) pathways [50] thereby exerting its neuroprotective effect.

The JAK-STAT signaling pathway is a chain of interactions between protein cells and is involved in many cellular processes that communicate information from chemical signals outside of a cell to the cell nucleus, resulting in the activation of genes through transcriptional processes [126]. The neuroprotective effect of G-CSF is manifested by activating the anti-apoptotic pathway via the JAK/STAT3 signaling, and it does so by suppressing the pro-apoptotic mediators and upregulating anti-apoptotic mediators via binding to its receptors (G-CSFR) in neurons. Moreover, G-CSF increases the activation of STAT3 pathway in glial cells together with increased cIAP2 expression which is a member of the inhibitor of apoptosis protein (IAP) family, subsequently regulating the activity of both initiator (caspase-9) and effector caspases (caspase-3 and -7) in ischemic mouse models [40, 94]. It can directly activate the JAK/STAT3 pathway as well [33, 93, 127], thereby promoting neurogenesis. Pim-1 increases cell survival through the regulation of bcl-2 proteins and that its upregulation after HI is enhanced by G-CSF treatment. Its expression is said to be paralleled to STAT3 expression, suggesting an association between the two in ischemic pathology [33, 94]. G-CSF also increased expression of STAT3 in the penumbra mediated by G-CSFR [128]. In short, these substrates and/or proteins are regulated by G-CSF and its receptor along the JAK/STATS signaling pathway following HI injury, which in turn favors neuroprotection.

The PI3K/AKT pathway is a key signaling pathway that participates in various cellular processes such as cell proliferation, survival, differentiation, and apoptosis [129, 130]. G-CSF enhances neurogenesis and neuroblast migration after stroke by regulating the PI3K/Akt pathway as well as modulates the NMDA receptor of glial cells exposed to PLS-induced brain damage [68]. The nuclear factor-kappa B (NF-κB) is a central transcriptional factor that is regulated and activated by Akt accompanied by activation of the inhibitor of κB (IKB) kinase (IKK) [131]. Activation of NF-κB, especially the canonical pathway, triggers the production of pro-inflammatory cytokines (PICs) and nitric oxide (NO) [132]. Phosphorylated Akt by G-CSF inactivates the canonical NF-κB pathway and inhibit the production of PICs and NO, further ameliorating neuroinflammation. Moreover, G-CSF phosphorylates Akt leading to downstream inactivation of GSK-3ß that will ultimately decrease apoptosis. G-CSF as well reduces adherens (VCAM-1 and ICAM-1) and increases tight junction (claudin 3 and 5) protein expression levels via the G-CSFR/PI3K/Akt/GSK-3ß signaling pathway [50].

The mammalian target of rapamycin/p70 ribosomal S6 protein kinase (mTOR/p70S6K) pathway has been implicated in neurogenesis as well, by decreasing the expression of PICs [133], as well as increasing IL-10 expression [134]. mTOR acts as a molecular system integrator to support organismal and cellular interactions with the environment [135], which regulates cellular metabolism, growth, and proliferation through two protein complexes, mTORC1 and mTORC2. mTOR activity is also thought to upregulate the translation of synaptic mRNAs via 4E-BP and S6K [136] by facilitating neuronal plasticity and activity [137]. Previous studies have demonstrated a decreased activation of S6K by rapamycin, an mTOR inhibitor, as well as loss of S6K function leads to increased astrocyte death in ischemic models, while G-CSF treatment increases S6K expression levels [138, 139], driving cellular functions as S6K phosphorylates and activates several substrates that promote mRNA translation initiation and other cellular processes [140]. Our previous study has shown that treatment with G-CSF decreases inflammatory mediators and apoptotic factors, attenuating neuroinflammation and neuronal apoptosis via the mTOR/p70S6K signaling pathway, which represents a potential target for treating HI induced brain damage in neonatal HIE [74].

The PI3K/AKT/mTOR pathway is an intracellular signaling pathway important in regulating the cell cycle. Therefore, it is directly related to cellular quiescence, proliferation, cancer, and longevity. PI3K activation phosphorylates and activates AKT, localizing it in the plasma membrane [52, 141]. G-CSF can upregulate brain-derived neurotrophic factor (BDNF) which in turn induces autophagy through the PI3K/Akt/mTOR pathway [2]. The neurotrophic nuclear transcription factor phosphorylated cAMP-responsive element-binding protein at serine 133 (*p*CREBSer-133) serves an important role in neurological regulation of ion channel function, neuronal differentiation and maturation, and the processes of learning and memory. This transcription factor is decreased during HI insult, while treatment of G-CSF was shown to significantly increase the expression of *p*Raf-*p*ERK1/2-*p*CREBSer-133 pathway in neonates exposed to perinatal hypoxia [61]. One study has demonstrated that G-CSF treatment inhibits steroidogenesis through activation of the JAK2/PI3K/PDE3B signaling pathway by reducing the levels of cAMP expression in HI-induced brain injury [142]. Activation of ERK has been shown to be neuroprotective, both in adults and neonatal brain injury, while MAPK p38 is best known for transduction of stress-related signals, regulation of inflammatory gene production, and NF- κB recruitment to selected targets, and both ERK and MARK p38 are regulated by G-CSF [37]. G-CSF downregulates the activation of the phosphorylated JNK and c-jun pathway in the cerebral ischemia-reperfusion rats model [44].

Thus, G-CSF plays diverse roles in neonatal HIE through diverse signaling pathways. Modi et al. [45] summarizes the steps and effect of G-CSF across the signaling pathways that have been implicated in neonatal HI injury either through inhibition or upregulation and phosphorylation of their substrates thereby eliciting neuroprotection.

## Conclusion

There are numerous studies that have shown the important role G-CSF plays in neurodegenerative diseases, ischemic stroke, and traumatic brain injury both using in vivo and ex vivo models. Recent research has specifically focused on its neuroprotective effect in neonatal HIE with both positive and mixed results. Indeed, G-CSF exerts a pivotal role in the control of immune response and acts as an anti-inflammatory cytokine, preventing an overactivation of monocytes and lymphocytes by reducing the release of pro-inflammatory cytokines as well as stabilizing the BBB and modulating neuroinflammation; inhibits pro-apoptotic mediators; enhances concentration of neurotrophic factors and facilitates bone marrow cell mobilization thereby driving neurogenesis; increases local VEGF expression necessary for vascular angiogenesis; upregulates Ang-1 that reduces vascular solute permeability; and contributes to vascular maturation and BBB stabilization. Despite these progresses being made, there are still few experimentation models of neonatal HIE and G-CSF’s neuroprotectiveness either directly or through signaling pathways, and extrapolation of adults’ stroke models is challenging due to the evolving neonatal brain. Also, there are mixed results about the effectiveness of G-CSF in improving HI-induced brain injury in neonates, specifically in the area of initiation of treatment, dosage, route of administration, and duration of treatment; this calls for more in-depth research to elucidate its underlying mechanisms and pertinent role in neuroprotection, as well as its long-term effect in neurological and behavioral outcomes.

Recent studies have focused on combinational treatment in neonatal HI brain injury and that it is more effective than G-CSF monotherapy. Therapeutic hypothermia has also been advocated for with other agents such as EPO, melanin, xenon, stem cells, and anticonvulsants with promising results. As most of these agents have controversial effectiveness in improving HI-induced brain injury and most of them are under clinical trials for efficacy and safety, at present, it appears that combination of these therapeutic agents with TH could be the promising intervention strategies to treat newborns suffering from HIE, while awaiting the outcomes of both preclinical and clinical trials that are under investigation.

In this review, we highlighted recent researches in the role of G-CSF in regulating cytokines and inhibition of apoptotic mediators, as well as promoting neurogenesis and angiogenesis thereby enhancing cell survival and proliferation, and modulation of inflammatory responses in the injured neonatal brain through activation of multistep signaling pathways.

## Data Availability

Not applicable.

## Abbreviations

AIP: Apoptosis-inducing factor
Akt: Protein kinase B
Ang-1: Angiopoietin-1
BAD: Bcl-2-antagonist of cell death
BAX: Bcl-2-associated X protein
BBB: Blood-brain barrier
BLC-2: B cell lymphoma 2
BDNF: Brain-derived neurotrophic factor
cAMP: Cyclic adenosine monophosphate
CC3: Cleaved caspase-3
CNS: Central nervous system
EE: Enriched environment
EEG: Electroencephalogram
EPCs: Endothelial progenitor cells
EPO: Erythropoietin
FL: Fms-related tyrosine kinase 3 ligand
FLK-1: Fms-like tyrosine kinase 1
GABA: Gamma-aminobutyric acid
G-CSF: Granulocyte-colony stimulating factor
G-CSFR: Granulocyte-colony stimulating factor receptor
GDNF: Growth-derived neurotrophic factor
GSK-3β: Glycogen synthase kinase-3 beta
HI: Hypoxia ischemia
HIE: Hypoxic ischemic encephalopathy
HIF-1α: Hypoxia inducing factor-1 alpha
HSCs: Hematopoietic stem cells
hUCB: Human umbilical cord blood
IAP: Inhibitor of apoptosis protein
ICAM1: Intracellular adhesion molecule 1
IL-1β: Interleukin-1 beta
iNOS: Inducible nitric oxide synthase
JAK: Janus kinase
MAPK: Mitogen-associated protein kinase
MHC: Major histocompatibility complex
MMPs: Matrix metalloproteinases
MMP-9: Matrix metalloproteinase-9
MOMP: Mitochondrial outer membrane permeabilization
mTOR: Mammalian target of rapamycin
NF-κB: Nuclear factor-kappa B
NMDA: N-methyl-d-aspartic acid
NO: Nitric oxide
NVU: Neurovascular unit
PI3K: Phosphatidylinositol 3 kinase
PICs: Pro-inflammatory cytokines
PDKI: Pyruvate dehydrogenase isoenzyme
P70S6K: P70 ribosomal S6 protein kinase
RCT: Randomized control trial
SAEs: Severe adverse effects
SCF: Stem cell factor
STAT: Signal transducer and activator of transcription
SVZ: Subventricular zone
TBI: Traumatic brain injury
TH: Therapeutic hypothermia
TNF-α: Tumor necrosis factor alpha
TRAIL: TNF-related apoptosis-inducing ligand
UCB: Umbilical cord blood
UC-MSCs: Umbilical cord mesenchymal stem cells
VCAM1: Vascular cell adhesion molecule 1
VEGF: Vascular endothelial growth factor
